# Developmental Regression of Motor Ability in the Patient With Interruption of the Aortic Arch: Juvenile Idiopathic Arthritis Associated With Deletion of 22q11.2 Syndrome

**DOI:** 10.7759/cureus.106352

**Published:** 2026-04-03

**Authors:** Yu Matsuo, Hirofumi Saiki, Kunihiko Miura, Mikiya Endo

**Affiliations:** 1 Pediatrics, Iwate Medical University, Shiwa, JPN; 2 Pediatric Cardiology, Iwate Medical University, Shiwa, JPN; 3 Pediatrics, Iwate Prefectural Miyako Hospital, Miyako, JPN

**Keywords:** 22q11.2 deletion, arthritis, interruption of aortic arch, motor, regression

## Abstract

While developmental delay is often associated with 22q11.2 deletion syndrome and congenital heart diseases, developmental regression is rarely observed. We report the case of a one-year-old girl with 22q11.2 deletion syndrome whose motor development regressed after radical repair of an interrupted aortic arch and ventricular septal defect. Despite reasonable achievement of the initial motor milestones, she stopped crawling and was unable to pull herself to stand at one year and six months of age. No signs of suppressed cardiac function were observed, and residual coarctation was acceptable. Finger swelling coupled with limited knee extension was a clue to the suspicion of complicating juvenile idiopathic arthritis (JIA). Since our patient was refractory to the treatment with prednisolone and methotrexate, adalimumab was introduced, after which joint symptoms disappeared immediately without developing adalimumab-mediated cardiac dysfunction. Then, somatic growth as well as her motor development were regained without joint destruction. Although reported cases of JIA related to 22q11.2 deletion syndrome are primarily teen years, JIA became a source of developmental regression due to early onset in this case. Since early diagnosis and treatment are critical to prevent joint damage, physicians who participate in the medical care of patients with congenital heart diseases and 22q11.2 deletion syndrome should suspect JIA in case of motor developmental delay.

## Introduction

Developmental retardation is often observed in patients with repaired congenital heart disease, which can be attributed to specific circulatory properties, malnutrition associated with corrective surgeries, and psychosocial abilities associated with genetic background. The development of patients with 22q11.2 deletion syndrome is often slow yet steadily growing [1]. Here, we present a patient with 22q11.2 deletion syndrome who had regressed motor development after one and a half years of radical surgery for an interrupted aortic arch (IAA), which was attributed to juvenile idiopathic arthritis (JIA). While cellular immunodeficiency is frequently observed in cases of 22q11.2 deficiency syndrome, the development of JIA is also a significant complication. Although this condition is rarely observed in early childhood, its presence may manifest as developmental delay or regression. Therefore, when abnormalities in motor function are observed in children with 22q11.2 deficiency syndrome, the possibility of complicating JIA should be considered among the differential diagnoses.

## Case presentation

A one-year and nine months old girl visited us for a routine checkup after radical surgery for an IAA and ventricular septal defect (VSD). Her guardian stated concern about halted motor development for more than seven months after the establishment of a sitting position.

The patient was delivered at 40 weeks of gestation, with a body weight of 3,278 g and Apgar scores of eight and nine at one and five minutes, respectively. No cardiac anomaly was detected by an obstetrician prenatally. Although she did not exhibit distress at birth, she was referred to us because of a heart murmur. We diagnosed IAA complex type B and started continuous prostaglandin E1 infusion. Due to the rapid development of pulmonary high flow, bilateral pulmonary arterial banding was preceded, and total cardiac repair, including aortic reconstruction and VSD closure, was performed on the 19th day after birth. The patient was discharged from the hospital uneventfully. Owing to the characteristic cardiac anatomy and general appearance, we performed chromosomal analyses coupled with fluorescence in situ hybridization, which identified her as having 22q11.2 deletion syndrome. At three months of age, she developed mild aortic stenosis and recurrence of coarctation of the aorta; thus, a catheter intervention was planned. The pressure gradient at the stenotic sites was 17 mmHg, which remained unchanged after percutaneous angioplasty and was followed up on an outpatient basis. At the age of one year and two months, she was able to crawl forward but did not acquire the ability to pull up to stand thereafter. As her coarctation was mild and no symptoms of heart failure were observed, cardiac follow-up was planned in six months.

**Figure 1:**
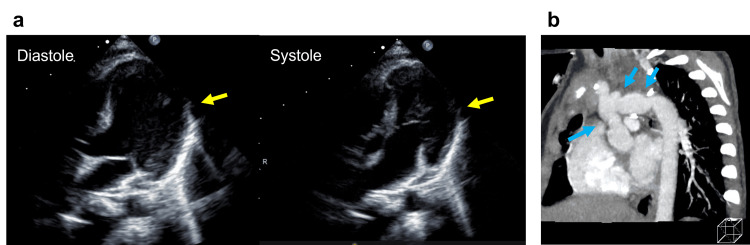
Left ventricular motion and shape of the aortic arch a. The left ventricular long axis view in diastole (left) and systole (right) is shown. The ventricular motion is satisfactory, and no apparent focal dyskinesis is observed. Mild left ventricular hypertrophy is identified (yellow solid arrows). b. The computed tomography angiogram of the aortic arch is shown. While the aortic arch has multiple stenotic lesions (blue solid arrows), the pressure gradient of non-invasive pressure measurements between upper and lower extremities is less than 10 mmHg.

At the next clinical visit at one year and nine months, her mother stated that the patient was unable to pull up to stand yet, and importantly, she became unable to crawl from the age of one year and six months. Her cardiac function was regarded as acceptable, as the blood flow velocity of the aortic arch was less than 3.0 m/second and the ventricular ejection fraction was satisfactory, despite mild left ventricular hypertrophy (Figure 1).

**Figure 2:**
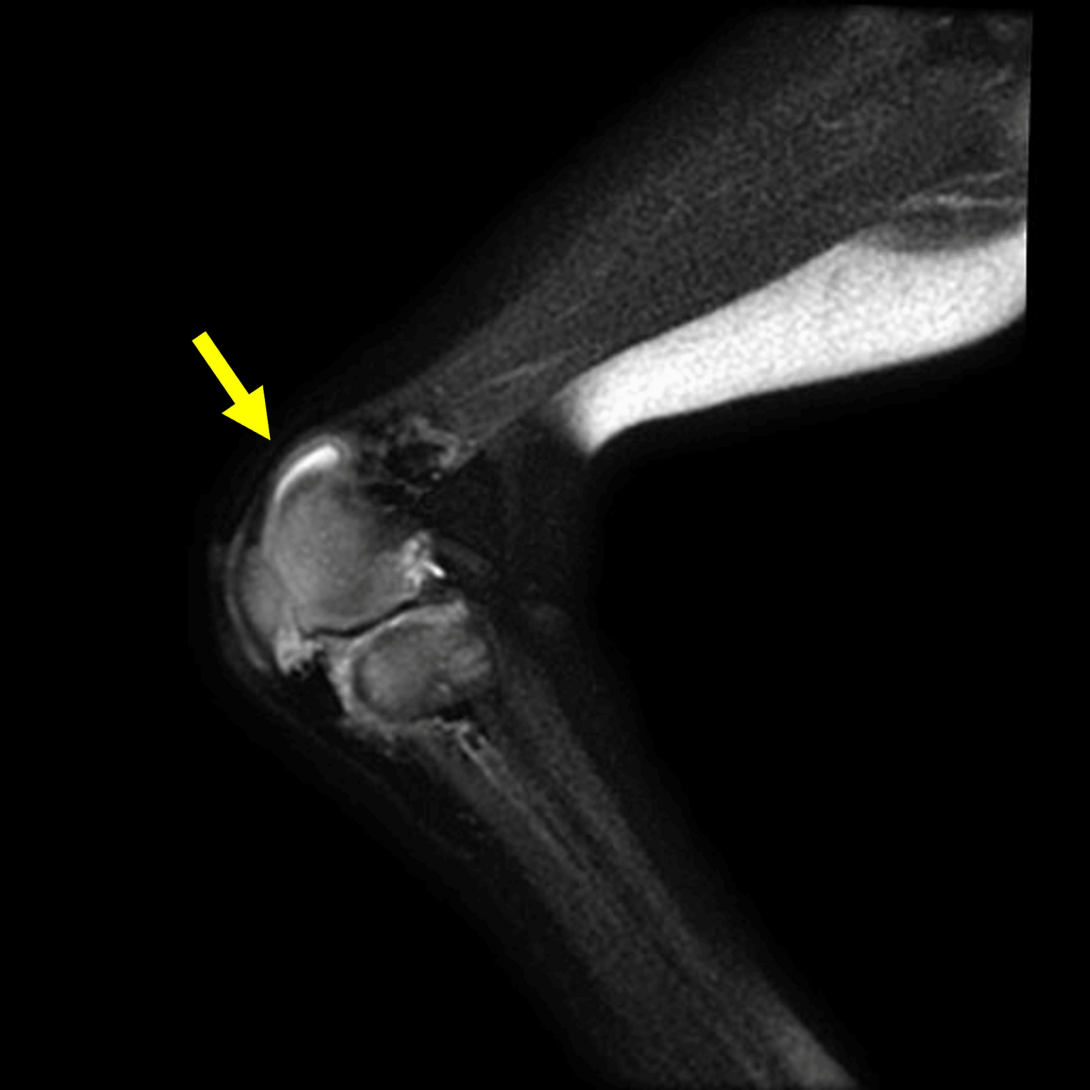
Magnetic resonance imaging of the knee joint The magnetic resonance imaging (T2-weighted image) of the right-side knee joint was shown (yellow arrow). The enhanced T2 signal of the synovial membrane indicated inflammation, suggesting a possible diagnosis of juvenile idiopathic arthritis. Similar findings were also identified in the left knee joint.

Consistent with the cardiac evaluation, no manifestation of impaired perfusion in the lower extremities was observed. The physical examination implied her fingers were swelling and edematous, which might be prominent in the morning. Although a congenital anomaly of the bilateral knee joint could not be ruled out, the patient’s knee joint showed limited movement with a range of -45° of extension to 150° of flexion. The magnetic resonance imaging (MRI) revealed a high-signal lesion on T2 imaging, suggesting synovitis (Figure 2).

Although she was afebrile and no apparent skin lesion was observed, the swollen fingers in the morning, coupled with the MRI findings, suggested the possibility of JIA. She remained afebrile; however, blood examination revealed a mild elevation of C-reactive protein, and she was often irritable, resisted sitting, and frequently assumed a supine position; thus, we consulted a rheumatologist at the age of two years. Initially, she was grumpy, and it was difficult to obtain physical findings of the joints. It looked as if there was no joint mobility bilaterally. The serum C-reactive protein level remained elevated, and the markedly elevated serum IgG level of 2589 mg/dl, which was markedly high for her age (reference value for one to four years old: 316-1148 mg/dL), indicated a persistently lasting inflammation. The matrix metalloproteinase-3 level was also markedly high, suggesting joint destruction due to rheumatic diseases (Table 1).

**Table 1:** Laboratory test Abnormal values are highlighted in bold.

Laboratory test	Value	Reference range
Blood cell count		
White blood cell count	12.0	4.2-18.8 × 10³/µL (two years old)
Hemoglobin	11.0	11.5-16.0 g/dL
Platelet count	435	150-450 × 10³/µL
Coagulation		
Prothrombin time	14	10-13 seconds
Prothrombin time-international normalized ratio	1.0	0.9-1.1
Activated partial thromboplastin time	37	26-38 seconds
D-dimer	2.0	<1.0 µg/mL
Biomarkers		
C-reactive protein	4.86	<0.30 mg/dL
Ferritin	137	5-120 ng/dL
Matrix metalloproteinase-3	183.2	17.3-59.7 ng/mL
B type natriuretic peptides	30.9	<18.4 pg/mL
Immune markers		
Immunoglobulin A	297	70-250 mg/dL
Immunoglobulin M	124	46-260 mg/dL
Immunoglobulin G	2589	870-1700 mg/dL
Complement C3	159	80-140 mg/dL
Complement C4	31	11-34 mg/dL
50% hemolytic complement activity	<60.0	<60 /mL
Auto antibodies		
Anti-nuclear antibody	1:40	<1:40
Rheumatoid factor	6	<15 IU/mL
Anti-double strand deoxyribonucleic acid antibody	2.7	<6.0 IU/mL
Anti-Smith antibody	2.2	<10.0 IU/mL
Anti-Sjögren's syndrome type A antibody	<1.0	<7.0 IU/mL
Anti-Sjögren's syndrome type B antibody	2.7	<7.0 IU/mL
Anti-ribonucleoprotein antibody	<2.0	<3.5 IU/mL
Anti-cyclic citrullinated peptide antibody	0.7	<4.5 IU/mL

**Figure 3:**
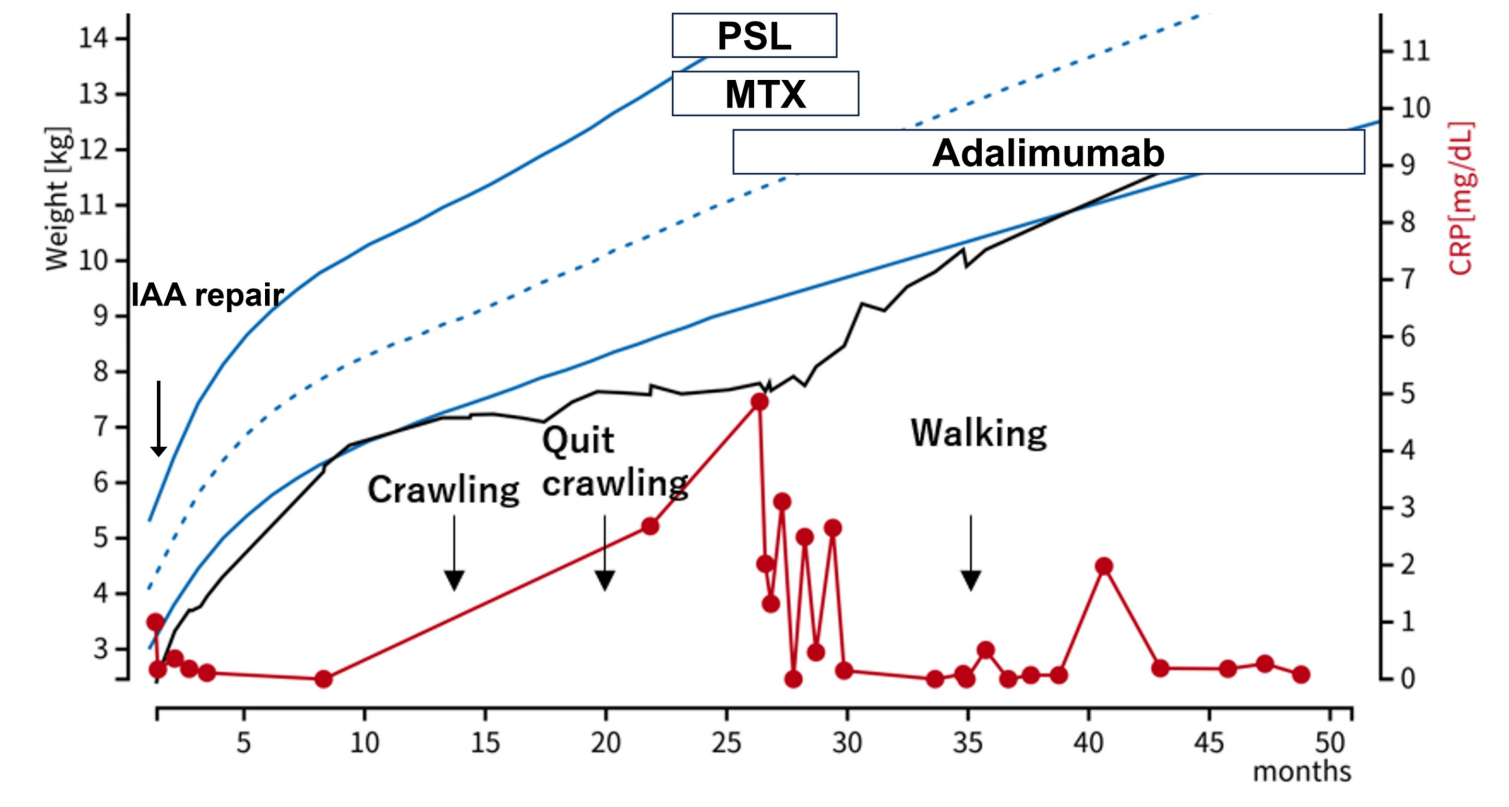
Clinical course The clinical course of this patient was shown. Body weight growth and motor development were regained after treatments. Black solid line: body weight. Red solid line: serum levels of C-reactive protein. Blue dotted and solid lines: reference range of body weight (mean ± 2 SD) in Japan. Abbreviations: IAA: interruption of the aortic arch; MTX: methotrexate; PSL: prednisolone Image was created using Microsoft PowerPoint (Microsoft® Corp., Redmond, WA).

Close inspection of the joints identified multiple joints swelling, including the majority of fingers of bilateral hands, bilateral patellar and foot joints, and the right sternoclavicular joint. No signs of infection, malignant diseases, or other sources of arthritis were detected based on cultures and laboratory findings, and lasting multiple joint swellings for more than six weeks led to the diagnosis of JIA based on the diagnostic criteria. Coupled with MRI findings, the laboratory findings of rheumatoid factor (RF)-negative and antinuclear antibodies (ANA) borderline-positive categorized her JIA as the polyarticular type. Even a month after starting oral prednisolone and methotrexate, the serum C-reactive protein (CRP) level remained high (Figure 3).

Accordingly, subcutaneous infusion of adalimumab was introduced, and she started to pull herself up to stand the next month, without developing cardiac dysfunction. The serum CRP and MMP-3 levels were within the normal range after 10 months of medication, and she was able to walk without difficulties. Indeed, the management of JIA was effective not only in promoting motor development but also in somatic growth (Figure 3). The patient required 20 months to walk after crawling; however, no joint destruction was observed.

## Discussion

A patient with 22q11.2 deletion syndrome developed lower limb motor dysfunction for more than a year after surgical repair of the IAA. Although the patient did not exhibit fever or symptoms of malnutrition, restricted joint movement coupled with swollen fingers was a clue to the diagnosis of JIA. Although the 22q11.2 deletion syndrome is often associated with a variety of comorbidities, including hypotonia, in which motor development might be affected, its potential association with rheumatic diseases remains underrecognized by cardiologists. Indeed, we were initially unable to isolate JIA as the most plausible source of motor development regression; however, the clinical course after therapeutic intervention confirmed a strong impact of JIA rather than 22q11.2 deletion itself or residual cardiac lesion. Homans et al. summarized orthopedic issues in their review and emphasized the importance of early recognition of JIA, particularly to the orthopedic surgeons [2].

In a cohort of 80 patients with 22q11.2 deletion, Sullivan et al. reported the prevalence of polyarthritis as 3.75% [3], which was approximately 50-150 times higher than the prevalence in the general population [2]. JIA is predominantly classified into subtypes, including systemic, oligoarticular, and polyarticular JIA, with or without positivity for serum RF. Some researchers questioned whether JIA that develops in patients with 22q11.2 deletion might be distinct from the typical JIA from the view of frequency of uveitis and positivity of RF [4]. Our patient was classified as polyarticular, RF-negative, and ANA borderline positive (articular type JIA), but indeed, no manifestation of uveitis, which is typical for the reported JIA in patients with 22q11.2 deletion. Not limited to the joint involvement, recent research clarified the lifetime prevalence of autoimmune diseases in patients with 22q11.2 deletion, as high as 10-30% [5], suggesting the importance of close observation.

As the chromosome 22q11.2 deletion syndrome is a haploinsufficiency of multiple genes, multiple systemic organs can be involved in developmental delay. While the very early motor development milestones, including rolling over and sitting alone, in patients with 22q11.2 deletion are comparable to those of their siblings, the acquisition of crawling, cruising, and walking abilities is markedly late, with a mean age of 10.9 ± 5.1, 14.3 ± 4.9, and 16.7 ± 4.6 months, respectively [1]. Interestingly, children nine to 15 years of age with 22q11.2 exhibited relatively comparable gross motor skills with those of community control subjects, although advanced motor skills, including physical balance, were compromised [6], rationalizing implementation of intellectual education and preservation of the musculoskeletal system. Regardless of multiple cardiac surgeries and residual aortic coarctation, our patient developed crawling at 14 months of age. Accordingly, based on a previous report [1], the patient was expected to be able to walk before two years of age. The inability to walk and even quit crawling, coupled with the recognition of finger swelling by her guardians, were important clues for the diagnosis of JIA, contributing to the prevention of joint destruction. Although the patient was refractory to the medications, including steroids and methotrexate, adalimumab was particularly effective in suppressing joint symptoms as well as improving somatic growth. Due to the bidirectional effects of TNF-α on the inflammatory cytokines, adalimumab might suppress cardiac function. Although we could not find previous reports on the use of adalimumab in small children with congenital heart disease and 22q11.2 deletion syndrome, no adverse impact on cardiac function was identified.

## Conclusions

Our case highlights the importance of considering systemic complications, including JIA, as potential causes of motor developmental delay or even regression in patients with 22q11.2 deletion syndrome. Although early detection of JIA is crucial for preventing joint destruction, developmental delays, which can be affected by both cardiac diseases and systemic properties influenced by genetic background, may hinder the identification of joint symptoms. Therefore, cardiologists should remain vigilant for the potential presence of a wide range of complications, including joint symptoms, which allows for early consultation with other specialists, including pediatric rheumatologists, ultimately enhancing the quality of life of affected individuals. Arthritis associated with 22q11.2 deletion syndrome may exhibit a distinct pathophysiology of JIA in unaffected patients, necessitating further case studies to deepen our understanding.
